# Suicidal thinking and behavior in young people at Clinical High Risk for Psychosis: Psychopathological considerations and treatment response across a 2‐year follow‐up study

**DOI:** 10.1111/sltb.13136

**Published:** 2024-10-19

**Authors:** Lorenzo Pelizza, Alessandro Di Lisi, Emanuela Leuci, Emanuela Quattrone, Derna Palmisano, Clara Pellegrini, Pietro Pellegrini, Giuseppina Paulillo, Simona Pupo, Marco Menchetti

**Affiliations:** ^1^ Department of Biomedical and Neuromotor Sciences “Alma Mater Studiorum” Università Degli Studi di Bologna Bologna Italy; ^2^ Department of Mental Health and Pathological Addictions Azienda USL di Parma Parma Italy; ^3^ Division of Pain Medicine, Department of Medicine and Surgery Azienda Ospedaliero‐Universitaria di Parma Parma Italy

**Keywords:** clinical high risk, early psychosis, follow‐up, outcome, suicide, treatment

## Abstract

**Introduction:**

Suicidal ideation has high rates among individuals at Clinical High Risk for Psychosis (CHR‐P). CHR‐P mental states are currently defined as attenuated psychotic symptoms, brief intermittent psychotic symptoms, or genetic risk and functioning deterioration syndrome. However, the relationship between psychotic experiences and suicidality in CHR‐P subjects is still not fully understood. Research emphasizes the need to address suicidality in CHR‐P individuals due to its incidence and severe socio‐economic impact. This study aimed to assess the baseline prevalence and 2‐year incidence rates of suicidal thinking and behaviors in an Italian CHR‐P sample, investigate the stability of suicidal ideation over 2 years, and examine its associations with treatment outcomes, sociodemographic characteristics, and clinical factors.

**Methods:**

CHR‐P participants were treated in an “Early Intervention in Psychosis” program and completed the PANSS and the GAF scale at baseline and every 12 months.

**Results:**

180 CHR‐P individuals were enrolled (92 with suicidal ideation [SI+]). SI+ subjects had a higher baseline prevalence of past suicide attempts. Over 2 years, a decrease in suicidal ideation severity was observed in the total group. Longitudinal improvement in disorganized symptoms was a key predictor of the decrease in suicidal ideation. Participants with a history of suicide attempts were more likely to attempt again.

**Conclusion:**

Addressing disorganization is crucial for suicide prevention in the CHR‐P population. Continuous risk monitoring and preventive actions are needed for those with past suicide attempts.

## INTRODUCTION

Elevated rates of suicidal thought and behavior are prevalent in individuals with psychotic disorders and extend to the wider population experiencing attenuated psychotic experiences and at‐risk mental states (Poletti et al., 2023). In this respect, the current paradigm of early psychosis identifies three main “Clinical High Risk for Psychosis” (CHR‐P) subgroups: (1) “Attenuated Psychotic Symptoms” (APS), (2) “Brief, Limited, Intermittent Psychotic Symptoms” (BLIPS), and (3) “Genetic Risk and Functioning Deterioration” (GRFD) syndrome (McGorry et al., 2006). Specifically, APS are the most common CHR‐P mental states, with an approximately 80% prevalence rate (Pelizza, Leuci, et al., 2024). Individuals with APS show mild, attenuated positive/disorganized symptoms that must be of sufficient severity/frequency to warrant clinical attention, must have been present at least once per week for the past month, and must have begun/worsened in the last year. APS also are not better explained by other mental disorders (e.g., affective disorders with psychotic features) and are not attributable to physiological effects of a substance or another medical condition. Finally, criteria for any psychotic disorder have never been met. BLIPS subjects have criteria for overt psychosis met for less than a week and ceased spontaneously (i.e., without antipsychotic medication). The GRFD syndrome is a state–trait condition combining a family history of psychosis in first‐degree relatives or schizotypal personality disorder in the patient with 30% drop in functioning or chronic low functioning in the past year.

Anyway, the nature of the link between suicidal thinking/behavior and CHR‐P mental states is still ambiguous. Psychotic experiences could directly trigger suicidal ideation via symptoms such as command hallucinations, or indirectly through common risk factors like depression or substance abuse (Yates et al., 2019). Notably, when confounding factors were considered in general population studies, the association between psychotic experiences and suicidal features persisted, albeit weaker, suggesting an independent correlation among adolescents and adults (Hielscher et al., 2018).

Disentangling the intricate relationship between psychotic experiences and suicidal tendencies is crucial for Early Intervention in Psychosis (EIP). Given that suicide is a leading cause of death among youths (with a 29.9% average prevalence of suicidal thought and a 9.7% lifetime prevalence of suicide attempts) (Evans et al., 2005), the importance of addressing this issue is clear. In this respect, also adolescents and young people at Clinical High Risk for Psychosis (CHR‐P) (McGorry et al., 2006) showed a significant prevalence of suicidal thought and behavior (Taylor et al., 2015), with severity and incidence being comparable to patients with First‐Episode Psychosis (FEP) (Pelizza, Poletti, Azzali, Garlassi, et al., 2020). This evidence underscores the urgency of understanding suicidality within CHR‐P individuals, its risk factors, and effective interventions, also considering the high socio‐economic impact of suicidality (Whitlock et al., 2014). Indeed, the increased suicide risk in CHR‐P populations is often a more immediate concern than the risk of psychosis transition (Bang et al., 2019; Hutton et al., 2011).

Recent empirical investigations explored other potential suicide determinants in CHR‐P individuals, yielding mixed results. Some authors reported the possible predictive role of depression severity, anhedonia, psychiatric comorbidity, impaired social functioning, and suspiciousness (Haining et al., 2021; Pelizza, Paterlini, et al., 2019; Poletti et al., 2022). Gender differences in the impact of childhood and adolescent trauma on suicidal ideation have also been observed (Salokangas et al., 2019), as well as significant associations with dysphoric mood and odd behavior (D'Angelo et al., 2017). Finally, the role of family history of nonpsychotic mental disorders and concurrent nonpsychotic mental disorder comorbidity (particularly, major depressive disorder, obsessive‐compulsive disorder, and substance use disorder) was also found (Pontillo et al., 2020). However, there is a need for more in‐depth prospective studies to thoroughly examine these clinical links to suicidality and to investigate the effectiveness of the specialized EIP intervention on suicidal risk (Wastler et al., 2023).

As for treatment response for suicidality in CHR‐P populations, evidence is very scarce. A study examining the impact of family treatments on clinical outcomes in CHR‐P adolescents found that family interventions could reduce subthreshold positive symptoms, but did not necessarily impact depressive symptoms, including suicidal ideation (Rinne et al., 2021). Furthermore, a systematic review of clinical trials (Calear et al., 2016) reported that while psychosocial interventions could reduce suicidal thought and self‐harm in young people without psychiatric diagnoses, their effectiveness varied widely, with school‐based programs showing particular promise.

Starting from this background, our study aimed: (a) to calculate baseline prevalence and 2‐year incidence rates of suicidal thought and behavior in an Italian CHR‐P population treated within a specialized EIP program, (b) to compare sociodemographic and clinical characteristics at baseline between CHR‐P individuals with and without suicidal ideation; and (c) to examine the longitudinal stability of suicidal ideation in the total CHR‐P sample across a 2‐year follow‐up period and any significant association of changes in suicidal thinking and behavior with treatment response and sociodemographic and clinical parameters.

## MATERIALS AND METHODS

### Setting and participants

All CHR‐P participants were sequentially recruited within the “Parma At‐Risk Mental States” (PARMS) program from January 2016 to December 2021. The PARMS program is a specialized EIP infrastructure diffusely implemented across all adolescent and adult mental health services in the Parma Department of Mental Health (Northern Italy) (Pelizza et al., 2023).

Inclusion criteria of this examination were: (a) to seek specialized mental health assistance; (b) age 12–25 years, and (c) to meet CHR‐P criteria as defined by the “Comprehensive Assessment of At‐Risk Mental States” (CAARMS) at the baseline assessment (i.e., Attenuated Psychotic Symptoms [APS], Brief Limited Intermittent Psychotic Symptoms [BLIPS], and “Genetic Vulnerability”) (Yung et al., 2005).

Exclusion criteria were: (a) past psychotic episode; (b) previous exposure to AP drug or current AP intake for a duration exceeding 4 weeks in the present illness episode, (c) known intellectual disability (IQ < 70); and (d) neurological or other medical disorder with psychiatric manifestations. Past use of AP medication was considered a proxy for past psychotic episode, consistently with the original CAARMS definition of psychosis threshold (Yung et al., 2005). A current AP prescription of less than 4 weeks was required to minimize pharmacological interference with baseline psychopathological assessment (Pelizza, Di Lisi, et al., 2024).

All participants and parents (if minors) provided their written informed consent for their participation in the study. This research obtained approval from the local ethics committee (AVEN Ethics Committee protocol n. 559/2020/OSS*/AUSLPR) and adhered to the principles outlined in the 1964 Declaration of Helsinki and its later amendments.

### Instruments

The *clinical assessment* encompassed the CAARMS, the “Positive And Negative Syndrome Scale” (PANSS) (Kay et al., 1987), and the “Global Assessment of Functioning” (GAF) scale (APA, 2016).

The *CAARMS* is a clinical interview designed to explore multiple aspects of attenuated psychopathology. Its “Positive Symptoms” subscale serves as the basis for defining both CHR‐P and psychosis criteria. CAARMS interviews were conducted by trained PARMS team members using the approved Italian version (CAARMS‐ITA) (Raballo et al., 2013). Regular CAARMS scoring workshops and supervision sessions were implemented to ensure good interrater reliability measures. Specifically, the CAARMS‐ITA showed to have good‐to‐excellent interrater reliability values (for both total and subscale scores: i.e., Intra‐Class Correlation [ICC] coefficient >0.75), as well as good‐to‐excellent internal consistency values (i.e., Cronbach's alpha coefficient >0.85) (Pelizza, Paterlini, et al., 2019). Finally, the CAARMS‐ITA also had good‐to‐excellent concurrent and construct validity values when its scores were compared to the PANSS scores and were obtained in patients with early psychosis in comparison with psychiatric controls (Pelizza, Azzali, et al., 2019).

In this examination, the presence of current suicidal ideation was detected using an item 4 cut‐off score of ≥3 in the Brief Psychiatric Rating Scale (BPRS) (e.g., “Have you felt that life wasn't worth living?”, “Have you thought about harming or killing yourself?”, “Have you felt tired of living or as though you would be better off dead?”) (Ventura et al., 1993). This cut‐off score indicated current suicidal ideation. The *PANSS* is a widely used interview for assessing psychopathology in psychosis, also in youths at illness onset (Pelizza, Leuci, Landi, Quattrone, et al., 2020; Poletti et al., 2020). As proposed by Shafer and Dazzi (2019), we considered five main psychopathological dimensions: “Disorganization,” “Negative Symptoms,” “Positive Symptoms,” “Resistance/Excitement‐activity,” and “Affect.” The *GAF* is a commonly used scale for evaluating clinical status and socio‐occupational functioning in individuals with severe mental disorders, including early psychosis (Poletti et al., 2021).

As for psychometric properties of BPRS, PANSS, and GAF scores, validation studies of the Italian versions of these instruments overall showed good‐to‐excellent reliability and concurrent validity measures in patients with psychosis (i.e., ICC coefficients >0.80, Cronbach's alpha values >0.80, and Speraman's ρ coefficients >0.40) (for details, see also Pancheri et al., 1995; Roncone et al., 1999; Lo Cascio et al., 2017).

Lastly, we completed a sociodemographic/clinical chart (collecting information on “Duration of Untreated Illness” [DUI]), new suicide attempt and self‐harm, functional recovery, and service disengagement. A suicide attempt was defined as potentially injurious, self‐inflicted behavior without a fatal outcome for which there was (implicit or explicit) evidence of intent to die (Silverman et al., 2007). Self‐harm behavior was intended as acts of deliberate self‐harm or intoxication with alcohol or drugs, but where there was no clear intention to die (Silverman et al., 2007).

All assessment instruments were administered both at baseline (T0) and every 12 months during the 2‐year follow‐up period (i.e., T1 and T2).

### Procedures

The initial DSM‐5 diagnosis was established through assessments conducted by trained PARMS team members, using the Structured Clinical Interview for DSM‐5 disorders (SCID‐5) (First et al., 2016). Following CAARMS interviews, CHR‐P individuals with current suicidal ideation (i.e., with a BPRS item 4 score of ≥3) were categorized in the CHR‐P/SI+ subgroup. CHR‐P participants without current suicidal ideation at presentation were included in the CHR‐P/SI‐ subgroup.

Within 3–4 weeks from baseline assessment, CHR‐P participants were assigned to a multi‐disciplinary team including a clinical psychologist, an early rehabilitation case manager, and a psychiatrist. In accordance with current official guidelines on EIP (RER, 2023; Schmidt et al., 2015), AP medication use at entry was reserved to CHR‐P individuals who (a) displayed rapid deterioration in daily functioning, (b) experienced a sudden escalation of full‐blown psychotic symptoms, (c) had an immediate risk of suicide or severe violence, or (d) failed to respond to psychosocial interventions. As a first‐line pharmacological treatment, low‐dose atypical AP drugs were administered (Pelizza, Poletti, Azzali, Paterlini, et al., 2020; Poletti et al., 2020).

Individual psychotherapy, family psychoeducation, and case management were provided to CHR‐P participants following specific intervention models (McFarlane et al., 2012; Van der Gaag et al., 2012). These approaches included at least 15 sessions of individual psychotherapy (Azzali et al., 2022), 10–12 sessions of family psychoeducation (Pelizza, Azzali, et al., 2019), and 24 sessions of early rehabilitation coordinated by a dedicated case manager over the first year of treatment (Ficarelli et al., 2021; Pelizza, Poletti, Azzali, Paterlini, et al., 2020), together with buster sessions in the second year according to specific patient's clinical needs and daily functioning.

Data on medication, psychosocial intervention, psychopathology, functioning, and outcomes were collected at baseline and throughout the 2‐year follow‐up period. Baseline prevalence rates and cumulative incidence rates of suicidal thinking and behavior were initially calculated. Then, we compared sociodemographic, clinical, and treatment parameters between CHR‐P subgroups at baseline. We also investigated any relevant association of suicidal thought with clinical and sociodemographic characteristics at entry. Finally, we examined the stability of suicidal ideation over the 2‐year period and any significant association of change in suicidal thinking and behavior with clinical parameters and treatment response.

### Statistical analysis

The data analysis was conducted using the Statistical Package for Social Science (SPSS) version 15.0 for Windows (SPSS Inc., 2010). All tests were two‐tailed, with *p* value significance set at 0.05. In between‐group comparisons, categorical variables were analyzed using the Chi‐square (*X* ^2^) test, while continuous variables using the Mann–Whitney *U* test. Spearman's rank correlation coefficients were calculated to examine the significant association of BPRS item 4 subscores with sociodemographic and clinical parameters both at baseline and during the 2‐year follow‐up period. Crude cumulative baseline prevalence and 2‐year incidence rates of suicidal thinking and behavior were initially examined. Then, we performed a Kaplan‐Meyer survival analysis to calculate their estimated cumulative risk rates across the follow‐up period. The Wilcoxon test for repeated measures was also performed to assess the longitudinal stability of BPRS item 4 scores in the CHR‐P total sample across the 2 years of follow‐up.

Binary logistic regression and multiple linear regression analyses with suicidal thinking and behavior scores as dependent variables and intensity of specialized PARMS intervention components, sociodemographic and clinical measures as independent variables were also carried out. In our longitudinal examinations, we specifically used the differences (deltas [Δ]) between T0 and T1 or T2 PANSS or BPRS item 4 scores as primary psychopathological parameters to examine overtime. Indeed, according to Ver Hoef (2012), the delta scores better describe longitudinal changes and temporal dynamics of psychosis psychopathology compared to T0, T1, and T2 single subscores.

## RESULTS

In this investigation, 19 (10.6%) CHR‐P participants had a history of suicide attempts and 92 (51.1%) experienced current (2‐week) suicidal ideation at entry (Table 1). Across the follow‐up, our findings revealed an increase in crude cumulative incidence of suicide attempt over time: that is, from 3.3% (*n* = 6) at T1 to 7.2% (*n* = 13) at T2. No completed suicide was found. Kaplan‐Meyer survival analysis results showed slightly higher estimated cumulative risks of suicide attempt (i.e., 5.9% at T1 and 8.5% at T2).

**TABLE 1:** Rates of suicidal thinking and behavior in the CHR‐P total group (*n* = 180).

Variable	
Baseline prevalence rate	
Previous suicide attempt	19 (10.6%)
Current suicidal ideation	92 (51.1%)
Cumulative incidence rate	
1‐year suicide attempt	6 (3.3%)
2‐year suicide attempt	13 (7.2%)
1‐year completed suicide	0 (0.0%)
2‐year completed suicide	0 (0.0%)
Estimated cumulative risk rate	
1‐year suicide attempt	0.059 (0.019)
2‐year suicide attempt	0.085 (0.023)

*Note*: Frequencies (and percentages) and cumulative risk estimates (and standard error) are reported.

Abbreviation: CHR‐P, Clinical High Risk for Psychosis.

### Baseline data

At presentation, the 92 CHR‐P participants having current suicidal ideation were classified as CHR‐P/SI+. The remaining 88 (48.9%) were included in the CHR‐P/SI‐ subgroup (no current suicidal ideation) (Table 2). Compared CHR‐P/SI‐, CHR‐P/SI+ individuals had a higher baseline prevalence of past suicide attempt. No other intergroup significant differences were found in terms of sociodemographic and other clinical features. As for between‐group comparisons on PANSS single item subscores, the CHR‐P/SI+ subgroup showed more severe levels in N2 “Emotional Withdrawal” (*z* = −2.321; *p* = 0.040), N4 “Passive Social Withdrawal” (*z* = −2.309; *p* = 0.042), and G6 “Depression” items (*z* = −2.799; *p* = 0.010) (see Supplementary Materials [Table S1] for details).

**TABLE 2:** Baseline sociodemographic and clinical comparisons between the two CHR‐P subgroups.

Variable	CHR‐P/SI+ (*n* = 92)	CHR‐P/SI− (*n* = 88)	*X* ^2^/z	*p*
Gender (males)	45 (48.9%)	45 (51.1%)	0.089	0.766
Ethnic group (white Caucasian)	81 (88.0%)	78 (88.6%)	0.015	0.901
Migrant Status	17 (18.5%)	11 (12.5%)	1.224	0.269
Civil status (single)	89 (96.7%)	86 (97.7%)	0.236	0.627
Living status (with parents)	86 (93.5%)	85 (95.6%)	0.998	0.231
NEET	27 (29.3%)	27 (30.6%)	0.235	0.876
Age (at entry)	19.55 ± 4.04	19.50 ± 3.53	−0.029	0.977
Education (in years)	11.26 ± 2.52	11.39 ± 2.37	−0.667	0.505
DUI (in weeks)	46.36 ± 50.91	46.40 ± 46.78	−0.460	0.646
Past hospitalization	13 (14.1%)	15 (17.0%)	0.291	0.590
Past specialist contact	38 (41.3%)	45 (51.1%)	1.750	0.186
Past attempted suicide	16 (17.4%)	3 (3.4%)	9.314	**0.002**
Family history of psychosis	30 (32.6%)	29 (33.0%)	0.002	0.961
Current substance abuse	12 (13.0%)	19 (21.6%)	2.305	0.129
CHR‐P subgroups				
APS	69 (75.0%)	71 (80.7%)	0.840	0.359
BLIPS	15 (16.3%)	15 (17.0%)	0.018	0.894
Genetic vulnerability	8 (8.7%)	2 (2.3%)	2.450	0.118
PANSS score				
Positive symptoms	12.89 ± 6.75	12.08 ± 6.75	−0.611	0.541
Negative symptoms	20.26 ± 7.43	18.21 ± 7.81	−1.485	0.138
Disorganization	15.78 ± 5.41	15.52 ± 5.31	−0.379	0.705
Affect	15.86 ± 5.00	14.86 ± 5.39	−1.057	0.290
Resistance/Excitement‐activity	7.60 ± 3.05	6.86 ± 3.38	−1.871	0.061
Total score	72.65 ± 16.11	68.39 ± 18.86	−1.609	0.108
GAF score	43.76 ± 8.00	49.77 ± 9.13	−1.462	0.144
Antipsychotic medication prescription	46 (50.0%)	46 (52.3%)	0.093	0.760
Antidepressant medication prescription	27 (29.3%)	16 (18.2%)	3.084	0.079
Mood stabilizer medication prescription	5 (5.4%)	12 (13.6%)	3.538	0.060
Benzodiazepine medication prescription	21 (22.8%)	20 (22.7%)	0.0001	0.987

*Note*: Frequencies (and percentages), means ± standard deviation. Chi‐squared test (*X* ^2^) and Mann–Whitney *U* test (*z*) values are reported. Bonferroni's corrected *p* values are reported. Statistically significant *p* values are in bold.

Abbreviations: APS, Attenuated Psychotic Symptoms; BLIPS, Brief Limited Intermittent Psychotic Symptoms; CHR‐P, Clinical High Risk for Psychosis; CHR‐P/SI−, CHR‐P individuals without current SI; CHR‐P/SI+, CHR‐P individuals with current SI; DUI, Duration of Untreated Illness; GAF, Global Assessment of Functioning; NEET, Not in Education, Employment, or Training; p, statistical significance; PANSS, Positive And Negative Syndrome Scale; SI, current Suicidal Ideation.

Considering only parameters found to be statistically significant in intergroup comparisons as independent variables, our binary logistic regression analysis results in the CHR‐P total group showed that baseline current suicidal ideation was exclusively predicted by past suicide attempts (Table 3).

**TABLE 3:** Binary logistic regression results on dichotomized baseline BPRS item 4 subscore (cut‐off of ≥3) by clinical parameters previously resulted statistically significant in the CHR‐P total group (*n* = 180).

Variables	B	SE	Wald	Df	*p*	OR	95% CI for OR lower upper	
Previous suicide attempt	1.543	0.818	3.566	1	**0.047**	0.235	0.043	1.061
PANSS N2 Emotional withdrawal	0.116	0.183	0.401	1	0.526	0.890	0.622	1.275
PANSS N4 Passive social withdrawal	0.099	0.150	0.435	1	0.509	0.906	0.674	1.216
PANSS G6 Depression	0.196	0.162	1.461	1	0.227	0.822	0.598	1.130
Constant	1.487	0.607	6.004	1	0.014	4.423	—	—

*Note*: Overall model fit test: *X* ^2^ = 12.904; *p* = **0.012**; overall percentage = 62.3%; associated strength: Cox‐Snell *R* ^2^ = 0.100; Nagelkerke *R* ^2^ = 0.134. Statistically significant *p* values are in bold.

Abbreviations: BPRS, Brief Psychiatric Rating Scale; CHR‐P, Clinical High Risk for Psychosis; PANSS, Positive And Negative Syndrome Scale; B, regression coefficient; SE, Standard Error; df, degree of freedom; p, statistical significance; OR, Odds Ratio; CI, Confidence Intervals.

### Longitudinal data

Across the entire 2‐year follow‐up period, we observed a significant longitudinal decrease in suicidal ideation severity levels in the CHR‐P total group (Table 4). This T0‐T2 reduction in suicidal thinking showed a significant positive association with the baseline presence of APS, as well as relevant positive correlations with longitudinal improvement in PANSS “Disorganization” and “Resistance/Excitement‐Activity” dimension scores (especially in terms of P2 “Conceptual Disorganization” [*ρ* = 0.210; *p* = 0.053], G13 “Disturbances of Volition” [*ρ* = 0.202; *p* = 0.043], and G14 “Poor Impulse Control” item subscores [*ρ* = 0.359; *p* = 0.001]) (see Supplementary Materials [Table S2] for details). Moreover, a relevant statistical trend was also observed in the negative correlation between this temporal decrease in current suicidal thoughts and past attempted suicide (*p* = 0.057).

**TABLE 4:** Longitudinal association between BPRS item 4 subscores and sociodemographic/clinical parameters in the CHR‐P total group across the 2‐year follow‐up period.

Variable	T0 (*n* = 180)	T1 (*n* = 175)	T2 (*n* = 153)	T0‐T1 *z* (*p*)	T1‐T2 *z* (*p*)	T0‐T2 *z* (*p*)
BPRS item 4 subscores	1.82 ± 1.93	1.66 ± 0.47	0.97 ± 1.46	−3.719 (0**.0001**)	−3.952 (0**.0001**)	−4.937 (0**.0001**)
**Variable (*n* = 153)**			**T0‐T2 BPRS item 4 subscore (ρ/z)**		***p***	
Gender			−0.055		0.501	
Ethnic group (white Caucasian)			0.103		0.203	
Migrant status			−0.134		0.098	
Age (at entry)			0.064		0.430	
Education (in years)			−0.063		0.439	
NEET			−0.178		0.076	
DUI (in weeks)			−0.064		0.434	
Past hospitalization			0.088		0.282	
Past attempted suicide			−0.155		0.057	
Past specialist contact			0.032		0.695	
Family history of psychosis			0.044		0.589	
Current substance abuse			−0.018		0.826	
CHR‐P subgroups						
APS			0.162		**0.046**	
BLIPS			−0.94		0.249	
Genetic vulnerability			−0.049		0.550	
T0 PANSS scores						
Positive symptoms			0.082		0.314	
Disorganization			0.063		0.532	
Affect			−0.109		0.277	
Resistance/Excitement‐Activity			0.079		0.433	
Total score			0.56		0.577	
T0‐T2 PANSS scores						
Positive symptoms			0.075		0.457	
Disorganization			0.219		0.**028**	
Affect			0.004		0.965	
Resistance/Excitement‐activity			0.268		0.**007**	
Total score			0.173		0.085	
T0‐T2 GAF score			−0.108		0.248	
Antipsychotic medication prescription			−0.048		0.552	
Antidepressant medication prescription			−0.048		0.553	
Mood stabilizer medication prescription			0.135		0.096	
Benzodiazepine medication prescription			0.006		0.943	

*Note*: Mean (standard deviation), Wilcoxon test (*z*), Spearman rank correlation (*ρ*), and Mann–Whitney *U* test (*z*) values are reported. Bonferroni's corrected *p* values are reported. Statistically significant *p* values are in bold.

Abbreviations: BPRS, Brief Psychiatric Rating Scale; CHR‐P, Clinical High Risk for Psychosis; DUI, Duration of Untreated Illness; GAF, Global Assessment of Functioning; p, statistical significance; PANSS, Positive And Negative Syndrome Scale; T0, baseline assessment time; T1, 1‐year assessment time; T2, 2‐year assessment time.

Linear regression analysis results (including PARMS treatment components and the clinical features found to be statistically significant in our previous examinations as independent parameters) showed that longitudinal decrease in suicidal ideation was specifically predicted by improvement in disorganization level over time (Table 5). In particular, disorganization effects on suicidal thinking exceeded effects for other psychosis parameters “to a statistically significant degree.” However, a relevant statistical trend in prediction was also shown by T2 equivalent dose of fluoxetine (*p* = 0.056). No association was found with antipsychotic treatment and psychosocial interventions provided within the PARMS protocol.

**TABLE 5:** Linear regression analysis results for T0‐T2 delta BPRS item 4 scores by specialized treatment components of the PARMS program and clinical parameters previously resulted statistically significant in the CHR‐P total sample across the 2‐year follow‐up period.

T0‐T2 BPRS item 4	B	SE	Β	*p*	95% CI		
					Lower	Upper	
Constant	2.075	1.044	—	0.050	0.001	4.150	*R* ^2^ = 0.230 F_[df = 13]_ = 1.976 *p* = 0**.032**
T0 equivalent dose of risperidone (mg/day)	−0.038	0.098	−0.057	0.699	−0.232	0.156	
T1 equivalent dose of risperidone (mg/day)	−0.134	0.119	−0.221	0.266	−0.371	0.104	
T2 equivalent dose of risperidone (mg/day)	0.211	0.152	0.271	0.168	−0.090	0.513	
T0 equivalent dose of fluoxetine (mg/day)	−0.003	0.003	−0.155	0.244	−0.008	0.002	
T1 equivalent dose of fluoxetine (mg/day)	−0.003	0.002	−0.194	0.204	−0.008	0.002	
T2 equivalent dose of fluoxetine (mg/day)	0.006	0.003	0.341	0.056	0.001	0.013	
T2 number of individual psychotherapy sessions	0.001	0.009	0.009	0.946	−0.017	0.018	
T2 number of family psychoeducation sessions	−0.008	0.015	−0.074	0.583	−0.039	0.022	
T2 number of case management sessions	0.003	0.002	0.124	0.223	−0.002	0.007	
Previous suicide attempt	−0.695	0.468	−0.152	0.142	−1.626	0.237	
T0‐T2 PANSS Resistance/Excitement‐activity	0.080	0.052	0.167	0.132	−0.025	0.184	
T0‐T2 PANSS Disorganization	0.088	0.031	0.315	0.**005**	0.027	0.149	
APS subgroup	−1.66	0.576	−0.030	0.774	−1.312	0.980	

*Note*: Statistically significant *p* values are in bold.

Abbreviations: 95% CI, 95% Confidence Interval for B; APS, Attenuated Psychotic Symptoms; B, unstandardized coefficient; BPRS, Brief Psychiatric Rating Scale; CHR‐P, Clinical High Risk for Psychosis; F, statistic test value for linear regression analysis; p, statistical significance; PANSS, Positive And Negative Syndrome Scale; PARMS, Parma At‐Risk Mental States; *R* ^2^, R‐squared or coefficient of determination; SE, Standard Error; T0, baseline assessment time; T2, 2‐year assessment time; *β*, standardized coefficient.

Finally, our binary logistic regression analysis results also showed that a history of past suicide attempt at entry was the only predictors of new attempted suicide across the follow‐up (Table 6).

**TABLE 6:** Binary logistic regression analysis results for new suicide attempt by specialized treatment components of the PARMS program and clinical parameters previously resulted statically relevant in the CHR‐P total sample across the 2‐year follow‐up period.

New suicide attempt (*n* = 13)	B	SE	Wald	Df	*p*	HR	95% CI for HR	
							Lower	Upper
Previous suicide attempt	3.690	0.926	15.883	1	**0.001**	0.025	0.004	0.153
T0 equivalent dose of risperidone (mg/day)	−0.154	0.264	0.339	1	560	1.166	0.696	1.955
T1 equivalent dose of risperidone (mg/day)	0.445	0.254	3.062	1	0.080	0.641	0.390	1.055
T2 equivalent dose of risperidone (mg/day)	0.005	0.300	0.001	1	987	0.995	0.552	1.792
T0 equivalent dose of fluoxetine (mg/day)	0.005	0.005	0.750	1	0.386	0.996	0.985	1.006
T1 equivalent dose of fluoxetine (mg/day)	0.004	0.005	0.609	1	0.435	0.996	0.985	1.006
T2 equivalent dose of fluoxetine (mg/day)	0.004	0.007	0.228	1	0.633	0.997	0.982	1.011
T2 number of individual psychotherapy sessions	−0.001	0.024	0.001	1	0.993	1.000	0.955	1.047
T2 number of family psychoeducation sessions	−0.043	0.048	0.820	1	0.365	1.044	0.951	1.148
T2 number of case management sessions	−0.001	0.008	0.002	1	0.968	1.000	0.985	1.016
T0‐T2 BPRS item 4	−0.254	0.208	1.494	1	0.222	1.289	0.858	1.936
Constant	−4.163	0.873	22.724	1	0.001	64.295	—	—

*Note*: Model summary: *X* ^2^ = 32.871; df = 11; ***p* = 0.001**; Cox & Snell *R* ^2^ = 0.194; Negelkerke *R* ^2^ = 0.440; overall percentage = 92.1%. Statistically significant *p* values are in bold.

Abbreviations: 95% CI, 95% Confidence Interval for OR; APS, Attenuated Psychotic Symptoms; B, unstandardized coefficient; BPRS, Brief Psychiatric Rating Scale; CHR‐P, Clinical High Risk for Psychosis; df, standardized coefficient; HR, Hazard Ratio; p, statistical significance; PANSS, Positive And Negative Syndrome Scale; PARMS, Parma At‐Risk Mental States; *R* ^2^, R‐squared or coefficient of determination, SE, Standard Error; T0, baseline assessment time; T2, 2‐year assessment time; *X* ^2^, Chi‐Squared test value.

## DISCUSSION

The clinical relationship between psychotic experiences and suicidal thinking and behavior remains complex and multifaceted. Psychotic symptoms, such as command hallucinations, may directly cause suicidal ideation. However, it is also possible that common underlying risk factors (including depression, anhedonia, and substance abuse) serve as indirect conduits linking psychotic features to suicidal outcomes. (Hor & Taylor, 2010; Yates et al., 2019).

In this research, a significant prevalence and incidence of suicidal thought and behavior was found in CHR‐P individuals. Indeed, at presentation, more than half (51.1%) of our participants experienced current suicidal ideation, and approximately one CHR‐P subject in 10 (10.6%) reported a past suicide attempt. Furthermore, the results of this investigation also showed an “alarming” trend: that is, across the 2‐year follow‐up period, the incidence rate of new attempted suicide grew from about 6% at T1 to 8.5% at T2, although no completed suicide was committed. Considering that suicide ranks among the leading causes of death in youths (Courtet, 2018), with those at CHR‐P typically experiencing higher rates of suicidal ideation and attempt, our findings emphasize a higher risk for suicide a suicidal vulnerability in this population, aligning with previous research. In this respect, evidence on relevant levels of suicidal thinking and behavior in CHR‐P adolescents and young adults (Haining et al., 2021; Taylor et al., 2015) strongly supports the idea that these subjects are not only at risk for psychosis but also, and perhaps even more, at risk for suicide. Therefore, paying close attention to suicidality in young CHR‐P individuals attending EIP programs is crucial for suicide prevention. Additionally, providing adequate training on this topic for general practitioners, health professionals working in emergency services, and mental health staff in generalist centers is essential. Our incidence rates of suicidal phenomena were similar to those reported in comparable Italian clinical sample of CHR‐P subjects and patients with FEP (Pelizza, Poletti, Azzali, Garlassi, et al., 2020; Pelizza, Leuci, Landi, Quattrone, et al., 2020; Pelizza, Poletti, Azzali, Paterlini, et al., 2020; Pelizza, Poletti, et al., 2019).

In this investigation, when the cohort was divided into two subgroups based on the presence or absence of current suicidal ideation, a relevant divergence emerged in terms of past suicide attempt. CHR‐P individual with current suicidal ideation (CHR‐P/SI+) showed higher rates compared to individuals without suicidal ideation (CHR‐P/SI‐). This finding suggests the crucial role of previous suicide attempts as a clinical indicator marker for the likelihood of current suicidal ideation in CHR‐P individuals upon their enrollment within specialized EIP programs. Consequently, from for effective suicide prevention, it is essential to assess the presence of current suicidal thinking in young CHR‐P individuals, especially those with a history of suicide attempt. Moreover, this emphasizes the presence of a consistent and extended risk of suicide among this young population which can easily and rapid progress from suicidal ideation to acts of self‐harm and may culminate in the ultimate and irreversible act committed suicide, particularly during periods of stressful events. It is therefore imperative and urgent to increase awareness among mental health professionals regarding this topic, emphasizing its importance for in public health prevention efforts. (Pompili et al., 2007).

As for PANSS item scores, the research results indicate that CHR‐P/SI+ subgroup experience more severe levels of emotional withdrawal, passive social withdrawal, and depression. These findings seem to highlight a specific psychopathological connection between current suicidal ideation and the baseline severity of emotional disturbances such as clinical depression and socio‐emotional withdrawal. This supports existing evidence that depression and anhedonia are important predictors for suicidal ideation in CHR‐P subject. (Poletti et al., 2023).

However, the study's binary logistic regression analysis results identified only a history of past suicide attempt as relevant predictor of current suicidal ideation at entry, reinforcing its potential clinical importance in identifying CHR‐P individuals at increased risk for suicide at the recruitment time within specialized EIP services. The absence of statistical significance for the PANSS depressive and negative items in our logistic regression analysis suggests the need for cautious interpretation of symptomatic correlations with current suicidal ideation and underlies the need for future studies on larger CHR‐P populations using more specific assessment instruments for negative symptoms depression in psychosis (such as the Brief Negative Symptom Scale or the Negative Symptom Inventory and the Calgary Depression Scale) (Addington et al., 1992; Kirkpatrick et al., 2011). Moreover, there is a need for an in‐depth investigation into the psychopathological mechanisms by which psychosis‐related symptoms may influence the risk of suicide, without necessarily serving as direct predictors.

Throughout the 2‐year follow‐up period, we documented a significant decrease in suicidal ideation in the CHR‐P total group, potentially reflecting a reduction in state‐risk factors and/or the effectiveness of our therapeutic interventions. Interestingly, this attenuation in the severity of suicidal thinking severity showed statistically significant positive associations with specific dimensions of psychopathology. In particular, we documented a notably psychopathological link with aspect of disorganization which may play a more critical role in suicidality than previously understood. Specifically, the results of our investigation showed statistically significant associations between suicidal ideation in CHR‐P individuals and reduction in disorganization severity (particularly in conceptual disorganization [P2] and disturbances of volition [G13]) over the two‐year follow‐up period. Additionally, we also found a statistically relevant mediation effect of disorganization on improvement in suicidal thinking over time using a path diagram analysis (Sobel test statistic = 4.832; Standard Error = 0.062; *p* = 0.022; point estimate = 0.388) (see Supplementary Materials [Figure S1] for details). In this respect, disorganization in thought and behavior is often associated with poor executive functioning (planning, decision‐making, and therefore impulse control). This reduction seems to be associated with improved cognitive and emotional regulation which allows better management of distress and impulsivity, which are key factors in suicidal behavior as shown by significant correlations between improvements in these disorganization subscores and reductions in suicidal thoughts.

The exact mechanism is not cleared yet, but reducing disorganization symptoms might reflect a more coherent thought processes, which could diminish reduce suicidal ideation by reducing distress felt by the patients and it could lead to better planning, decision‐making, and impulse control. It could help restore neural pathways, strengthening connections between the prefrontal cortex (crucial for cognitive control) and the amygdala, which handles emotional responses. (Miller & Cohen, 2001). Treating disorganization symptoms could be beneficial in reducing suicidality among CHR‐P, especially in early intervention by focusing on cognitive and functioning in CHR‐P individuals. For instance, cognitive therapies, which have shown promise in improving cognitive deficits in schizophrenia could be adapted to focus on disorganization in CHR‐P individuals (Eack et al., 2009) by using cognitive therapies or targeted psychosocial interventions designed to improve organizational and planning skills that can also help to improve social cognition and social interactions, therefore feelings of isolation or misunderstanding that could worsening suicidal thoughts. Also, mindfulness‐based interventions, which have been shown to improve cognitive and emotional regulation, could be beneficial in addressing disorganization symptoms (Chadwick et al., 2016).

Our findings underline the potential for using changes in disorganization as clinical marker of treatment response. It could be helpful in monitoring suicidal thoughts and in adjusting therapeutic strategies.

In conclusion, while the predictive role of past suicide attempts remains an important clinical focus in order to identify individuals who have the highest risk of suicide, our study show a new complex relationship between the psychopathological dimensions of disorganization and its impact on suicidal behavior showing how a more integrated treatment model that considers it could potentially be beneficial in early intervention setting. Future studies should continue to explore these relationships using bigger and more diverse CHR‐P populations and incorporating neuroimaging techniques to better understand the biological basis of disorganization and its link to suicidality (Palaniyappan & Liddle, 2012).

The simple positive correlation with improvement in the “Resistance/Excitement‐Activity” dimension, especially regarding poor impulse control, supports the importance of addressing impulsive tendency and behavior in suicide risk management (Gvion et al., 2015). Finally, a relevant statistical trend was also reported in the negative correlation between reduction in suicidal ideation and a history of past suicide attempts. Collectively, these psychopathological relationships highlight potential therapeutic targets within the broader scope of treatment for CHR‐P individuals.

As for the impact of treatments provided within the PARMS program and their potential beneficial effects on longitudinal changes in suicidal ideation across the 2‐year follow‐up period, our results did not reveal substantial effectiveness of the standard psychosocial interventions (i.e., individual psychotherapy, psychoeducational support for family members, and traditional case management) in the CHR‐P population. This suggests that while these interventions are crucial to overall mental health care and support (due to their specific treatment modules on positive, negative and depressive symptoms), they may not sufficiently impactful to directly influence suicidal thoughts in the short term for CHR‐P individuals. Therefore, specific psychosocial intervention packages on suicidal thinking and behavior in CHR‐P subjects are needed, especially in a preventive perspective.

However, the findings of this study also showed a potential beneficial effect of antidepressant treatment pharmacological in longitudinally mitigating suicidal ideation. This association, though not reaching a sufficient statistical significance, hints at a complex but potentially meaningful role of pharmacotherapy in contributing to influence suicidal thought. An implication is that while antidepressant therapy may contribute to managing suicidal ideation, the dynamics of its impact are intricate, necessitating further in‐depth investigation. This finding emphasizes the need for an integrated therapeutic strategy that combines both pharmacological and more specific nonpharmacological interventions. Such an approach should be highly personalized (“person‐tailored”), adapting over time to the individual's symptoms and needs, to effectively support and manage their mental health (and specifically suicidal thinking and behavior). Finally, our results confirm that an historical pattern of previous suicide attempts significantly predicts future suicidal behavior. This supports the notion that a history of attempted suicide represents a clinically relevant behavioral antecedent, lowering the threshold for potential new attempts together with other predisposing and/or triggering factors (such as reduced fear of death, family history of attempting suicide, hopelessness, and increasing anhedonia) (Poletti et al., 2023).

### Limitations

This investigation has noteworthy limitations. First, the detection of suicidal ideation relied solely on a single‐item assessment. Therefore, future studies using more specific tools to assess suicidal thinking and behavior (such as the “Columbia Suicide Severity Rating Scale”) (Posner et al., 2011) are needed.

Furthermore, the study's relatively small CHR‐P subgroup raises concern about low incidence rates in outcome parameters. Conducting further research with larger CHR‐P populations is thus needed to address these limitations effectively.

## CONCLUSION

The findings of this study underscore the complexity of addressing suicidal ideation and behavior in the CHR‐P population. They advocate for a personalized, comprehensive treatment approach that integrates pharmacological treatment with psychotherapeutic and psychosocial interventions. Additionally, the close link between past and future suicide attempts calls for vigilant, ongoing risk assessment and targeted preventive measures for those individuals with a history of attempted suicide. Overall, this confirms the critical need for early and sustained intervention efforts.

Finally, changes in specific psychopathological dimensions pointing towards the relevance of addressing symptoms like disorganization, avolition, and poor impulse control are also crucial as part of suicide prevention strategies. The significant relationship between improvements in these clinical features and reductions in suicidal ideation and behavior highlights potential therapeutic targets within the broader scope of treatment for CHR‐P individuals.

## FUNDING INFORMATION

This research received no specific grant from any funding agencies in the public, commercial or not‐for‐profit sectors. The “Parma AT‐Risk Mental States” (PARMS) program was partly financed through a special, treatment‐oriented regional fund: “Progetto Esordi Psicotici della Regione Emilia Romagna.”

## CONFLICT OF INTEREST STATEMENT

The authors declare to have no conflict of interests.

## ETHICS STATEMENT

This research obtained approval from the local ethics committee (AVEN Ethics Committee protocol n. 559/2020/OSS*/AUSLPR) and adhered to the principles outlined in the 1964 Declaration of Helsinki and its later amendments.

## PATIENT CONSENT STATEMENT

All participants and parents (if minors) provided their written informed consent for their participation in the study.

## Supporting information


Data S1.

## Data Availability

The data supporting the findings of this investigation are not publicly available due to privacy/ethical restrictions, but may be shared with the corresponding author upon reasonable request.
