# Supplementary material for: Suicidal thinking and behavior in young people at Clinical High Risk for Psychosis: Psychopathological considerations and treatment response across a 2‐year follow‐up study
Source: Suicide Life Threat Behav. 2024 Oct 19;55(1):e13136. doi: 10.1111/sltb.13136 (PMC11716345; doi:10.1111/sltb.13136)
Supplement: Supplementary file 1 — Data S1. [file SLTB-55-0-s001.docx]

Table S1 – Baseline PANSS item subscore comparisons between the two CHR-P subgroups.

| PANSS item | CHR-P/SI+  (n = 92) | CHR-P/SI-  (n = 88) | X^2^/z | p |
| --- | --- | --- | --- | --- |
| P1 Delusions  P2 Conceptual disorganization  P3 Hallucinatory behavior  P4 Excitement  P5 Grandiosity  P6 Suspiciousness/Persecution  P7 Hostility  N1 Blunting affect  N2 Emotional withdrawal  N3 Poor rapport  N4 Passive social withdrawal  N5 Difficulty in abstract thinking  N6 Lack of spontaneity/flow of conversation  N7 Stereotyped thinking  G1 Somatic preoccupation  G2 Anxiety  G3 Guilty feelings  G4 Tension  G5 Postural mannerisms  G6 Depression  G7 Motor retardation  G8 Uncooperativeness  G9 Unusual thought content  G10 Disorientation  G11 Attention poverty  G12 Lack of judgment/insight  G13 Disturbances of volition  G14 Poor impulse control  G15 Preoccupation  G16 Active social avoidance | 2.02±1.31  2.15±1.18  1.97±1.09  1.12±0.56  1.38±1.01  2.92±1.46  2.75±1.50  3.23±1.56  3.45±1.44  2.03±1.46  3.66±1.61  1.92±1.38  2.28±1.55  1.92±1.35  1.95±6.75  4.15±1.54  2.43±1.49  2.91±1.83  1.26±0.71  4.42±1.48  1.82±1.04  1.62±1.10  2.45±1.46  1.25±0.78  2.06±1.14  2.40±1.53  2.02±1.42  2.11±1.42  3.20±1.85  3.80±1.72 | 2.02±1.19  1.95±0.99  2.02±1.23  1.22±.0.86  1.29±0.73  2.81±1.25  2.26±1.47  2.64±1.38  2.86±1.44  2.05±1.48  2.98±1.77  1.98±1.26  2.29±1.46  1.93±1.14  2.09±1.61  4.10±1.56  2.16±1.24  2.91±1.82  1.40±0.95  3.71±1.41  1.83±1.08  1.60±1.06  2.64±1.36  1.28±0.81  1.91±.0.88  2.07±1.24  1.93±1.55  1.76±1.16  3.14±1.54  3.59±1.74 | -.226  -.688  -.087  -.212  -.106  -.343  -1.980  -1.086  -2.321  -.217  -2.309  -.785  -.192  -.513  -.020  -.060  -.921  -.170  -.778  -2.799  -.022  -.322  -.970  -.185  -.433  -1.089  -.665  -1.429  -.013  -.627 | .822  .492  .931  .832  .915  .731  .096  .072  **.040**  .828  **.042**  .433  .848  .608  .984  .952  .357  .865  .437  **.010**  .982  .747  .332  .853  .665  .276  .506  .153  .990  .531 |

Note. CHR-P = Clinical High Risk for Psychosis; SI = current Suicidal Ideation; CHR-P/SI+ = CHR-P individuals with current SI; CHR-P/SI- = CHR-P individuals without current SI; PANSS = Positive And Negative Syndrome Scale; p = statistical significance. Means ± standard deviation, and Mann-Whitney U test (z) values are reported. Bonferroni’s corrected p values are reported. Statistically significant p values are in bold.

Table S2 – Longitudinal associations between BPRS item 4 scores and PANSS item subscores that constitute the “Resistance/Excitement-Activity” and “Disorganization” dimensions in the CHR-P total sample across the 2-year follow-up period.

| T0-T2 PANSS item scores | T0-T2 BPRS item 4  subscore (ρ) | p |
| --- | --- | --- |
| T0-T2 Resistance/Excitement-activity  P4 Excitement  P7 Hostility  G8 Uncooperativeness  G14 Poor impulse control  T0-T2 Disorganization  P2 Conceptual disorganization  N5 Difficulty in abstract thinking  N7 Stereotyped thinking  G5 Postural mannerisms  G10 Disorientation  G11 Attention poverty G13 Disturbances of volition  G15 Preoccupation | .116 .182 .147 .359   .210 .163 .057 .051 .046 .186 .202 .151 | .249 .070 .144 **.001**   **.035** .103 .574 .612 .645 .063 **.043** .131 |

Note. CHR-P = Clinical High Risk for Psychosis; BPRS = Brief Psychiatric Rating Scale; PANSS = Positive And Negative Syndrome Scale; T0 = baseline assessment time; T2 = 2-year assessment time; p = statistical significance. Spearman rank correlation (ρ) values are reported. Bonferroni’s corrected p values are reported. Statistically significant p values are in bold.

Figure S1 – Mediation effect of disorganization on improvement in suicidal ideation across the 2-year follow-up period: path diagram analysis.

| B = -.125 (SE = .043)  p = .010  T0-T2 PANSS “Disorganization” dimension score  B = -.263 (SE = .084)  p = .011  B = .450 (SE = .065)  p = .009  T2 BPRS item 4 score  T0 BPRS item 4 score  Sobel test: test statistic = 4.832; SE = .062; p = .022; point estimate = .388 |
| --- |

Note – BPRS = Brief Psychiatric Rating Scale; PANSS = Positive And Negative Syndrome Scale; B = linear regression coefficient; SE = Standard Error; T0 = Baseline assessment; T2 = 2-year assessment time; p = statistical significance.
